# Improving recovery from traumatic spinal cord injury: Targeting remyelination *versus* white matter remodeling

**DOI:** 10.4103/NRR.NRR-D-24-01628

**Published:** 2025-06-19

**Authors:** Bethany R. Kondiles, Wolfram Tetzlaff

**Affiliations:** International Collaboration on Repair Discoveries, Department of Zoology, University of British Columbia, Vancouver, BC, Canada

The inter-related pathological cascades following a traumatic spinal cord injury (tSCI) disrupt multiple cell types and physiological processes. Subsequently, motor and sensory functions are disrupted by breakdowns in cellular interactions and circuitry. Therapeutic interventions seek to modify some aspects of the injury course to enable the re-establishment of functional circuitry. Interventions often target one cell type (e.g., promoting neuroprotection or neural regeneration) or one process (e.g., modulating inflammation, affecting astrocytic, microglial, or macrophage responses.) Many axons in the spinal cord are myelinated, and after injury oligodendrocyte death causes demyelination. Promoting remyelination of spared or new axons to re-establish conduction seems a logical choice as a therapeutic target. However, “remyelination” refers to a binary process: the presence or absence of myelin regeneration. “White matter remodeling” considers the plasticity of and interactions between the multiple cell types essential for signal conduction. As the field develops more combinatorial approaches, wherein interventions target multiple cell types and processes, incorporating the concept of white matter remodeling, as opposed to remyelination, considers how to re-establish the requisite cellular circuitry necessary for regaining function.

**Figure 1:**
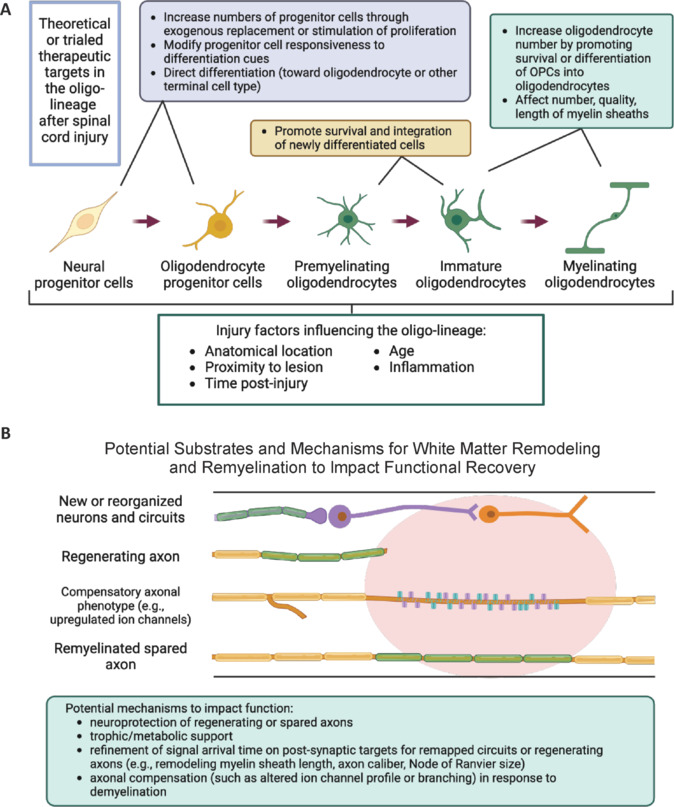
Potential therapeutic targets in the oligo-lineage and potential mechanisms of white matter remodeling post-injury. (A) A simplified diagram of the oligo-lineage illustrating potential therapeutic targets or strategies at each stage. Strategies range from well-studied and trialed (e.g., exogenous transplant), active areas of preclinical research (e.g., modifying progenitor cell responsiveness) to theoretical (e.g., modifying the number of myelin sheaths per oligodendrocyte or the length of myelin sheaths.) Other potential terminal fates for progenitor cells are not shown. The oligo-lineage can be further modified by external factors or indirectly affected by interventions targeting other cell types/processes (e.g., inflammation). These confounds complicate the search for interventions and interpretation of efficacy results. (B) A diagram detailing potential mechanisms by which white matter remodeling may promote functional recovery after injury. Newly generated or remapped neurons (in purple) may require new myelin (green) to adjust the timing of action potential propagation. Regenerating axons may require myelin for trophic and metabolic support. Stretches of demyelinated axons may induce compensatory phenotypes which can be targets for functional recovery. Remyelination of spared axons may promote neuroprotection and re-establish appropriate signal conduction. Created with BioRender.com. OPCs: Oligodendrocyte precursor cells.

In the central nervous system, oligodendrocyte precursor cells (OPCs) proliferate and differentiate into myelinating oligodendrocytes (**Figure 1A**). Myelinating oligodendrocytes provide trophic and metabolic support to axons. Myelin permits increases in action potential conduction velocity, and some data suggest myelination may tune the timing of signal propagation and arrival to post-synaptic contacts (for example, Steadman et al., 2020). The extent of chronic demyelination, the robustness of the remyelination response, and the necessity of remyelination after injury have been debated and studied as they relate to improving functional recovery after tSCI (reviewed in Pukos et al., 2019). Furthermore, studies in young rodents indicate that the large OPC population mounts a robust proliferative response after tSCI. As such, beyond serving as the source of oligodendrocytes, OPCs may play additional roles in the post-injury environment, with other terminal fates (reviewed in Duncan et al., 2020). Current research suggests that after traumatic injury, the formation of new myelin requires the differentiation of new oligodendrocytes, as post-mitotic mature oligodendrocytes have a limited capacity to form new myelin. Subsequently, interventions to target remyelination often entail promoting OPC proliferation (or exogenous transplant) for subsequent differentiation into oligodendrocytes (**Figure 1A**). However, recent research has increased our knowledge of the complexity and plasticity of the oligo-lineage, from progenitors to myelin, in both health and injury. Alongside studies of OPC differentiation to drive remyelination, so to must we consider the qualities and features of myelin sheaths and the axons they cover. Effectively targeting the oligo-lineage after injury requires consideration of the nuances of the lineage’s dynamics in the post-injury environment.

**3-month-old remyelination incompetent mice regain locomotor function after traumatic spinal cord injury:** To assess how differentiation of new oligodendrocytes and remyelination contributes to motor recovery, our laboratory knocked out these processes in a mouse model of tSCI. In this knockout model, the gene coding for a key transcription factor for the differentiation of oligodendrocytes, *Myrf*, is disrupted by exon 8 excision in OPCs a week prior to injury (Duncan et al., 2018). After injury, OPCs are unable to differentiate into oligodendrocytes, which inhibit remyelination. Extant myelin is unaffected. Following a moderate thoracic contusion at 3 months of age, knockouts regained locomotor function comparably to injured remyelination-competent controls (Duncan et al., 2018). Possible (and not mutually exclusive) explanations for these results include uninjured myelinated axons in the spared rim driving recovery or compensation through spared demyelinated axons which effectively conduct signal to re-establish locomotion. To investigate these possibilities, we reduced the amount of spared tissue by inducing a more severe injury and found that the remyelination knockout still did not differ from remyelination competent controls despite significantly fewer myelinated axons (Manesh et al., 2024). The remyelination-incompetent animals exhibited an altered ion channel expression pattern, wherein potassium channels and sodium channels were upregulated and colocalized across long stretches (> 50 µm) of axons. Taken together, these two studies suggest that 3-month-old mice might have compensatory mechanisms when remyelination is blocked that permit conduction through or around the lesion, enabling locomotor recovery.

**What can these data tell us regarding remyelination and functional recovery?** A perfunctory conclusion from these results is that remyelination is not an important target after tSCI. However, interpreting these studies alongside other current research instead suggests that the question “Does remyelination contribute to functional recovery after injury?” may be too simplistic. Current research provides evidence that the relationship between axons and the oligo-lineage is dynamic in the post-injury environment. As such, it may be more relevant to consider the oligo-lineage in entirety and determine which stage(s) are potential targets for interventions (**Figure 1A**) at which time points after injury.

**Figure 2:**
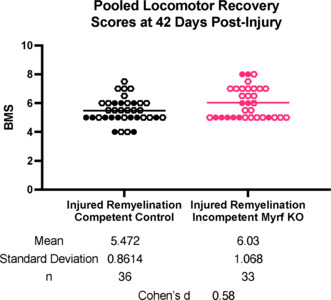
Comparison of locomotor recovery in remyelination incompetent mice and myelination competent controls after thoracic contusion Pooling data from Duncan et al. (2018) (hollow circles) and Manesh and Kondiles et al. (2024) (filled circles) demonstrates the moderate effect size of the knockout (KO) of remyelination on hind-limb stepping as assessed by the Basso Mouse Scale (BMS). At 42 days post-injury, remyelination is well underway in the injured control mice.

While rodent locomotor data should not be overinterpreted, it is curious that in our previous studies, remyelination incompetent mice trended towards better recovery on many of the assays (Duncan et al., 2018; Manesh et al., 2024). These experiments concluded at 42 days post-injury, when knockout animals have not replaced myelin lost post-injury, while newly differentiated oligodendrocytes in injured control animals are actively forming new myelin sheaths. We examined the pooled data assessing hind limb stepping using the Basso Mouse Scale from both our studies at this time point (**Figure 2**). A combined analysis shows a moderate effect size of the knockout as compared to injured controls (Cohen’s *d* = 0.58, control mean = 5.47 ± 0.86, *n* = 36, knockout mean = 6.03 ± 1.07, *n* = 33; *t*_(67)_ = 2.399, *P* = 0.019; **Figure 2**). The magnitude of these differences on the Basso Mouse Scale does not indicate substantial functional differences and the effect size is moderate. Nonetheless, it is intriguing to speculate if blocking oligodendrocyte differentiation and myelination after injury induced anatomical changes that could be amplified to produce functional improvements. Such changes could be mediated by a variety of mechanisms, such as a reduction in myelin-associated inhibitors of axon growth or altered axonal ion channel profiles. It remains unknown whether such compensatory mechanisms occur in humans. If so, they may prove a viable therapeutic target if they permit signal conduction across the relatively larger lesions. Validating these targets in humans may prove challenging, given that the duration of “chronic” demyelination remains unclear.

Indeed, the literature describes the oligo-lineage’s robust proliferative and remodeling response in young adult rodents following tSCI (for example, reviewed in Pukos et al., 2019; Duncan et al., 2020; Pukos et al., 2023). It remains to be seen if comparable responses also occur in the heterogeneous population of humans that sustain tSCI. If so, it is possible that spared or regenerating axons are quickly myelinated, with protracted remodeling and refinement extending for months after injury. Aberrant myelination is associated with axon pathology and remyelination in demyelinating diseases (for example, Bando et al., 2015). Furthermore, during development, the deposition of abnormal myelin requires microglia-dependent refinement (Djannatian et al., 2023). Regarding the trend of slightly improved locomotor function in remyelination knockout animals, one intriguing possibility is that early remyelination serves a role besides functional recovery. Remyelination may protect neurons against demyelination-mediated apoptosis (Duncan et al., 2024). Subsequently, it may be advantageous for the injured nervous system to quickly remyelinate to protect spared or newly generated axons. Protracted myelin refinement may reflect remodeling of myelin sheath length or axon coverage to tune conduction speed as the post-injury environment evolves over time (**Figure 1B**) In mouse models, the 2–3 mm lesion may not leave axons vulnerable enough to cause chronic demyelination mediated apoptosis, but rapid myelin deposition may play more vital neuroprotective roles in the larger lesions in humans. These possibilities illuminate the complexities and unknowns of targeting remyelination after tSCI. If the post-injury progression of a robust proliferative and remodeling response described in rodents also exists in humans, a therapeutic intervention to promote remyelination would require temporal precision. Poorly timed interventions to promote remyelination may reach ceiling effects if they coincide with spontaneous remyelination.

**Is there a critical window to promote remyelination after tSCI?** Based on data largely gathered from tSCI in adolescent rodents, acutely after injury OPCs proliferate followed by the deposition of new myelin in the following weeks-months (reviewed by Pukos et al., 2019). Studies assessing metformin and clemastine, pharmaceutical interventions that promote OPC differentiation, describe critical windows for efficacy. Only acute metformin administration after tSCI in 6–8 week-old mice improves functional recovery; animals receiving delayed administration did not differ from injured untreated controls (Gilbert et al., 2023). In these animals, metformin also induced sex-dependent reductions in microglial activation and stimulation of neural progenitor cells. In young (15-week-old) rabbits given demyelinating lesions, chronic clemastine administration promoted oligodendrocyte differentiation and reduced lesion volume, but exhausted progenitor pools and increased pro-inflammatory microglia (Cooper et al., 2024). Short-term administration (either acutely or delayed) failed to promote oligodendrocyte differentiation or reduce lesion volume. While these findings do not arise from tSCI models, they corroborate the findings from Gilbert et al describing critical windows for administration and effects on other cellular populations. Thus, the timing of interventions and the depletion of the OPC population are potential modifiers of efficacy in remyelination efforts that stimulate OPC differentiation.

**Remyelination *versus* white matter remodeling:** These studies highlight the importance of considering how white matter remodeling interacts with the injury course to promote functional recovery after injury. Furthermore, they illustrate the importance of considering the oligo-lineage in entirety. For example, differentiation and myelination is an energetically expensive and inefficient process, as most newly differentiated cells do not fully mature and die shortly after differentiation (reviewed by Hughes and Stockton, 2021). Should interventions also consider promoting immature and premyelinating oligodendrocyte survival? Furthermore, how do interventions that target the oligo-lineage interact with and complement ongoing neural and inflammatory processes in the post-injury environment (**Figure 1B**)? Neural circuits degenerate post-injury and interventions attempt to drive neural regeneration. The formation and maintenance of a myelin sheath is a plastic process that adapts to neural activity and the local environment. In rats, driving activity in the injured spinal cord induces shortened myelin sheaths and axonal branching (Kondiles et al., 2023). This myelin remodeling, wherein the structure of a myelin sheath, such as its length, is modified by neural activity, suggests that myelin can be modified by non-remyelinating interventions after injury. One intervention may target multiple forms of plasticity, or functional recovery might require a combinatorial approach. If, for example, an electrical stimulation intervention drives axonal branching, what is the appropriate timing for an intervention to promote OPC proliferation and differentiation to provide a large pool of oligodendrocytes to myelinate these branches? Furthermore, growing evidence implicates the inflammatory environment and microglial state on oligodendrocytes and myelin (for example, McNamara et al., 2023).

Post-tSCI, the deposition and refinement of myelin occurs in a dynamic environment of pro- and anti-inflammatory cascades, reactive astrocytes, and neuronal/axonal degeneration and plasticity. Interventions have the potential to further modify these inter-connected processes. These responses are affected by time post-injury as well as age at the time of injury. The fact that much of our knowledge regarding these phenomena arises from well-controlled studies in young rodents adds additional layers of complexity. Care must be taken when interpreting these data, as they fail to recapitulate the heterogeneity of the human populations sustaining tSCI. Combinatorial approaches and interventions may be necessary after tSCI to modify multiple targets and processes, and these approaches may need to be tailored to the individual and their injury. As hypothetical examples, an elderly individual may require early pro-differentiating interventions to promote neuroprotectant remyelination alongside interventions to dampen inflammatory responses. A younger individual receiving electrical stimulation to drive neural regeneration after injury may benefit from initial dampening of OPC proliferation and differentiation to promote compensatory axonal plasticity, followed by pro-OPC survival interventions to drive myelination of the newly generated axons. These hypothetical examples are not meant to be specific treatment plans, but rather are intended to illustrate how combinatorial dynamic and precise interventions could be adjusted to an individual’s injury course.

Trying to understand the contribution of the oligo-lineage to functional recovery after tSCI is made increasingly challenging by the multiple pathological cascades, the heterogeneity of the human population with tSCI, and the temporal dynamics of the oligo-lineage after injury. The necessity of myelin for axonal survival and action potential propagation supports the need for further research to discern the specific context in which targeting the oligo-lineage will promote functional recovery.

*We thank Dr. Greg J. Duncan for (Oregon Health & Science University, USA) the use of his data and his valuable feedback on this manuscript. Figure 1 was created in BioRender. Tetzlaff, W. (2025) https://BioRender.com/d06y619; https://BioRender.com/h19t597.*

*This work was supported by Grant 3195 from Paralyzed Veterans of America Research Foundation (to BRK). WT holds the Edie Ehlers Chair in spinal cord injury.*
